# Aggression and Justice Involvement: Does Uric Acid Play a Role?

**DOI:** 10.3390/brainsci15030268

**Published:** 2025-03-02

**Authors:** Alan C. Logan, Pragya Mishra

**Affiliations:** 1 Nova Institute for Health, Baltimore, MD 21231, USA; 2 Department of Law, Central University of Allahabad, Prayagraj 211002, India; pragya@allduniv.ac.in

**Keywords:** aggression, uric acid, microbiome, neuroinflammation, neuromicrobiology, neurolaw, legalome, forensic science, violence, antisocial behavior

## Abstract

The search for biological markers that can be reliably linked to aggression and antisocial behavior has been central to the work of biological criminology. One such marker, uric acid, has long been suspected to play a causative role in promoting anger, irritability, aggression, and violence. Here, in this perspective article, we revisit some of the historical interest in uric acid as a compound relevant to brain and behavior, and reflect these early accounts off emergent scientific research. Advances in brain sciences, including neuropsychiatry and neuromicrobiology, have allowed for a more sophisticated understanding of potential mechanistic pathways linking uric acid with cognition and behavior. The updated science suggests that some of the early ideas surrounding uric acid and criminology had credibility. The available research strongly suggests that uric acid, as a potential biomarker of risk, is worthy of further research and close scrutiny. Informed by emergent gut–brain–microbiome research, we argue that certain aspects of early-to-mid-20th-century biological criminology were prematurely abandoned. From a legalome perspective, further advances surrounding uric acid and other gut–brain biomarkers can aid in shaping more humane, scientifically grounded policies that recognize the interplay between biology and environment.

## 1. Introduction

“*The study and treatment of crime should be as individualized as is the study and treatment of disease. Confinement in the name of punishment is completely futile, worthless to society, unjust to the criminal, and utterly wrong*”

Louis Berman, MD, 1931 [1]

Historically, the quest to identify biological markers of criminal behavior, especially those indicative of causation rather than mere association or consequence, has been wrought with problems. For example, early efforts at identifying criminal phenotypes were rooted in racism and colonial logic [2]. These ideas filtered into the 20th century eugenics movement, which included compulsory sterilizations in correctional facilities and behavioral modification via aversive conditioning, the latter under the thin veil of ‘rehabilitation’ [3]. By the 1970s, biomedical criminology was best known for its folly, pseudoscience, and significant social harms [4]. However, integrative advances in neurosciences, biological psychology/psychiatry, microbiome sciences, nutritional medicine, psychoneuroimmunology, and microbiome sciences, to name a few, have led to a much more sophisticated understanding of biophysiology and behavior [5,6,7]. Much of this work has occurred external to criminology, a professional discipline that still tends to operate within a social science silo [8].

The rapidly growing field of neurolaw attempts to translate advances in brain science into the practical aspects of legal decisions and policy making [9]. The intersection of brain science and the law is a contentious area, one that forces difficult questions surrounding free will, blameworthiness, and moral responsibility [10]. In the popular press, neurolaw is synonymous with neuroimaging and the ways in which major signs of neuropathology (e.g., brain tumor) might influence behavior [11]. However, neurolaw pertains to both structure and function, and includes the entire exposome—that is, the total lived experiences (i.e., “exposures”—everything from heavy metal exposure to physical and/or psychological trauma) interacting with genes and the nervous system, over time. Neurolaw incorporates omics technologies and includes the microbiome [12]. Advances in brain sciences, including neuropsychiatry and neuromicrobiology, allow for a retrospective analysis of some of the 20th century claims (and preliminary findings) made by those who viewed criminology through a biological lens.

Here, in this perspective article, we focus on uric acid, a chemical formed by the metabolic breakdown of purine nucleotides. As discussed below, high blood levels of uric acid have long been suspected to play a causative role in irritability and aggression. Drawing from the PsycINFO, PubMed, and Google scholar databases, we examine contemporary scientific research, reflecting it off earlier discussions and findings found in Google Books, *NewsBank*, and Ancestry’s Newspapers.com. While ours is not a systematic review, search terms such as “uric acid + aggression” reveal that advances in mechanistic pathways linking uric acid to cognition and behavior, including the diet–microbiota–gut–brain axis [13], allow for the argument that uric acid is worthy of scrutiny as a target compound in neuropsychiatry. We also suggest that certain aspects of early-to-mid-20th-century biological criminology, including the brain-related implications of dietary factors and how these impair glucose metabolism, hormones, and endocrine factors [14,15,16], were prematurely abandoned.

## 2. Uric Acid and Behavior—Early History

“*Again, it has appeared to me that the irritability and bad temper of uric-acidemia, and the cerebral* [changes] *it brings about, may account for a certain number of murders*”

Alexander Haig, MD, 1892 [17]

Links between elevated uric acid and gout, an inflammatory condition caused by the deposition of urate crystals in joints, were established in the mid-1800s. However, in his book, *Uric acid as a factor in the causation of disease* (1892), London-based physician Alexander Haig argued that uric acid was a causative factor in many other conditions beyond gout, including hypertension and mental disorders [17]. Haig contended that uric acid increased psychological distress and irritability, while lowering the threshold for aggression and even violence.

Despite the popularity of the book, which was rereleased in several editions and translated in multiple languages, Haig’s claims were largely ignored by the medical community [18,19]. One exception was the well-known physician and social reformer Havelock Ellis. The modern research, to be discussed in the sections below, suggests Ellis was over the target in his 1904 writings on the relationships between uric acid and so-called ‘gouty men of genius’: “*These gouty men of genius have frequently been eccentric, often very irascible*—*choleric is the term applied by their contemporaries*—*and occasionally insane*” [20]. Here, we can note that the historical use of the term choleric temperament inferred “*pride and the lust of power, anger and irritability, hatred, revenge, and jealousy*” [21].

In the early 1930s, endocrinologist Louis Berman argued that much of criminal behavior is rooted in endocrine dysfunction. Berman’s three-year study of 250 inmates at the Sing Sing Penitentiary demonstrated significant endocrine disturbances among the prison population (compared to non-incarcerated controls) [22]. Although Berman’s study emphasized differences in thyroid, pituitary, thymus, and adrenal function, there were two observations within the routine blood tests that escaped discourse. The results showed that 32% of the inmates had high blood uric acid (unspecified normal range) compared to 5% of the non-incarcerated controls. In addition, 48% of the inmates were reported to have low blood sugar (unspecified normal range) compared to 12% of the controls [22].

Berman placed the uric acid findings in a table and made no reference to them in the body of his lengthy article, and the reader is left to ponder the relevancy of the endocrinologist’s findings [22]. Nevertheless, at least two other studies in correctional facilities supported Berman’s findings. The first, a 1974 study in the Morris County, New Jersey, at an adult jail, showed that inmates (*n* = 30) had higher levels of uric acid compared to non-incarcerated controls, and that among the inmates, those with a history of violence had the highest uric acid levels [23]. The second, involving 106 men admitted to Connecticut’s state forensic hospital for crimes of violence, showed that higher scores on the Overt Aggression Scale were associated with higher uric acid levels [24]. Support for the idea that uric acid influences behavior was also found in rare cases of purine metabolism disorders; in what is now known as Lesch–Nyhan syndrome, researchers noted that a genetically mediated enzyme deficiency results in excess blood uric acid and behavioral disturbances that include aggression and self-harm [25].

In the 1960s, several studies indicated that uric acid might be a marker of drive and determination in the world of business. For example, among 100 male business executives, uric acid levels were found to be higher than controls drawn from the general population, and within the ranks of the executives, those in the top echelon (vs. mid-level and junior-level executives) had the highest uric acid levels [26]. Similar links between higher blood uric acid levels and aspirations for high social standing were also found among male students [27,28], and between higher blood uric acid levels and drive (measured as “a life in which they are constantly working at top speed”), leadership (measured as a “greater interest in manipulating people than things”), and self-reported pride (related to perceptions of personal achievement) among male university professors [29].

These studies generally presented high uric acid levels as a potential marker of success and achievement, with inferences that gout, an inflammatory pathology associated with elevated uric acid, was most likely a product of the biochemistry of success and/or its associated lifestyle, one which leans toward expensive uric-acid-forming foods. This fed into the centuries-old narrative that gout is a disease of the wise and powerful—an affliction of the “great kings, emperors, generals, admirals, and philosophers” [30]. There was little, if any, discussion that males might have found themselves at the upper rungs of social hierarchy through excessive acts of hostility and aggression. In other words, there was no connection to Berman’s findings in the Sing Sing penitentiary.

Amid attempts to link uric acid with ‘success,’ there was also little critical examination of the question that was assumed to capture ‘drive’ (“constantly working at top speed”) and how it bears significant resemblance to measures of hyperactivity [31]. It could also be argued that measures of ‘leadership’ (answered as having an interest in “manipulating people”) in 1960s research were capturing aspects of Machiavellianism [32]. One group of Canadian researchers provided hints at the darker side of uric acid. Working with Kindergarten boys (aged 3 to 5) in 1969, the researchers did not find hypothesized connections between uric acid and intelligence tests. They did, however, note positive correlations between serum uric acid and aggressiveness, measured via a ‘Pugnacity Index’ [33].

While there was some lingering interest in links between uric acid and anger [34] and personality features (e.g., extraversion) [35] in the latter part of the 20th century, attention in psychobiology research waned. One older study involving three healthy adults supported the idea that uric acid is a chemical of risk and excitement; researchers drew ten blood samples over the course of 72 min while the participants relaxed and then either gambled for money in a poker game or played checkers (without financial involvement) for 24 min. The results indicated that once gambling commenced, unlike the relaxation phase or checkers gameplaying, uric acid levels were significantly elevated [36]. However, clear physiological mechanisms linking uric acid with aspects of behavior, personality, and/or social status were sparse. One enduring hypothesis, first presented by Louis Berman in 1932, proposed that uric acid held cortical-stimulating properties due to its structural similarity with other known psychostimulants (e.g., caffeine, theobromine, and theophylline) [37].

## 3. Contemporary Research

Perhaps due to the enormity of the crisis of non-communicable diseases, including brain-related diseases and disorders, the early 2000s witnessed significant growth in the understanding of the biological basis of diseases, as well as biology–environment interactions [38]. Advances in molecular biology, omics technologies, and neuromicrobiology resulted in a paradigm shift in brain sciences [39]. During this era, it became clear that uric acid is not merely a harmless waste product of concern only to clinicians treating gout. Rather, elevated uric acid levels were linked to multiple non-communicable diseases, including type II diabetes, hypertension, cancer, and cardiovascular and renal diseases [40,41]. Researchers also reported long-term elevations in uric acid among adults with worsened physical and mental health in response to a significant psychological trauma (i.e., major earthquake) [42]. Together, this led to a renewed interest in the biobehavioral aspects of uric acid, and links to depression, anxiety, and bipolar disorder emerged [43,44]. Using better designed studies, researchers scrutinized previous links to personality and suggestions that uric acid is a marker of ‘drive,’ finding that higher levels of uric acid are associated with impulsivity (disinhibition) and excitement seeking [45,46,47], and lower levels of conscientiousness and agreeableness [48]. Also in the early 2000s, it was noted that allopurinol, a drug that limits the formation of uric acid through the inhibition of the enzyme xanthine oxidase, reduces aggression in patients with neurologic dysfunction [49], dementia [50], and schizophrenia [51]. Allopurinol was also reported to be effective in the treatment of acute mania in adults with bipolar disorder [52,53].

In more recent years, higher blood levels of uric acid have been noted in patients with substance use disorders [54]; adults with antisocial personality disorder [55], borderline personality disorder [56], and attention deficit hyperactivity disorder [57]; and hospitalized patients with psychiatric disorders [58]. Researchers have also reported an inverse association between uric acid and cognitive function among middle-aged men and women in the general population [59]. It has also been noted that attenuated variants of Lesch–Nyhan disease, resulting in genetically mediated elevations in uric acid, are associated with a continuum of neurocognitive abnormalities [60]. Recent case reports from the Battle Creek Veterans Affairs Medical Center have also supported the use of allopurinol for refractory aggression in a variety of neuropsychiatric conditions [61]. In the pre-clinical realm, the experimental manipulation of uric acid (through disruption of the urate oxidase gene) indicates that uric acid promotes exploratory behavior, risk-taking (time in the open arms of the elevated plus maze), and novelty-seeking [47]. As mentioned earlier, several human studies have shown that higher levels of uric acid appear to be a product of psychological stress [62,63], while strong social support is linked to lower uric acid levels [64].

In a prospective study that more directly examined uric acid and aggression, researchers gathered baseline information on self-reported levels of aggression and urinary uric acid levels among urban youth (*n* = 84, average age 13), following up 18 months later. The aggression measure queried on the frequency with which a subject fought, hit, shoved, threatened to hit, or threw something at someone to hurt them over the previous 30 days. The results at follow-up showed that a higher excretion of uric acid (captured via 12 h overnight urine collection) predicted more frequent last-month aggression [65]. Finally, in a group of patients (*n* = 99) with affective disorders, higher blood uric acid levels were associated with verbal aggression on the Modified Overt Aggression Scale (MOAS) [66]. This is important because verbal aggression on the MOAS has been noted to be a predictor of future violent behavior [67]. Higher serum uric acid levels have also been linked to anxiety in adolescents living with obesity [68]. In a recent study of 100 in-patients who had been admitted while in a state of acute agitation, uric acid levels were found to be significantly higher in individuals suffering from mania than those with non-affective psychosis, and such elevations were positively correlated with validated symptom severity scales [69].

## 4. Emerging Mechanisms

At this point, the precise mechanisms linking elevated uric acid to impulsive, aggressive, and antisocial behavior have not been fully elucidated. Pre-clinical studies show that the stimulation of the hypothalamic–pituitary–adrenal (HPA) axis and the administration of stress hormones (e.g., epinephrine, norepinephrine) increase blood levels of uric acid [70,71,72]. Stress increases the activity of xanthine enzymes, which metabolize purines in the formation of uric acid [73]. In turn, uric acid plays a role in stress reactivity and the further regulation of stress physiology [74,75].

At the neural level, purines regulate neurotransmission [76], and it appears that increased uric acid levels lead to a reduction in adenosinergic transmission; this translates into a problem of a lack of inhibition and neuronal excitability [77]. Uric acid can influence both pre- and postsynaptic neurons, as well as specific receptors on glial cell membranes [78]; the reach of uric acid includes dopamine, gamma-aminobutyric acid, glutamate, and serotonergic neurotransmission [79]. Animal studies also demonstrate that uric acid passes freely through the blood–brain barrier, and chronic elevation leads to neuroinflammation and cognitive dysfunction [80,81]. Indeed, the ability of hyperuricemia to induce chronic low-grade inflammation is likely to be a primary unifying mechanism that helps to explain, at least partially, the role of elevated uric acid in a host of non-communicable diseases, including neuropsychiatric conditions [82,83].

Human studies employing brain imaging indicate that uric acid can influence areas of the brain involved in emotional regulation [84,85]. Human research also confirms that higher blood levels of uric acid are associated with higher levels in the cerebrospinal fluid, supporting the idea that even if there are blood–brain barrier (BBB) limitations on uric acid, the barrier function varies among individuals [86]. The BBB describes a specialized microvasculature inclusive of wedged endothelial cells and various transporters, efflux pumps, and cellular components that work to exclude potentially harmful substances from the brain and central nervous system [87]. The most obvious result of compromised BBB integrity is neuroinflammation, which in turn compromises normal cognition and behavior [88]. Emerging research demonstrates that the integrity of the blood–brain barrier is significantly impacted by various stressors [89], and assumptions regarding the access/exclusion of select chemicals cannot be made in clinical and non-clinical groups [90]. In animal models, a more porous blood–brain barrier is associated with increased aggression [91], and human research (using cerebrospinal-fluid-to-albumin ratio) indicates increased blood–brain barrier permeability in violent offenders [92].

Neurons operate at high metabolic rates and are highly dependent upon the optimal functioning of mitochondria, the so-called cellular ‘powerhouses’ that are responsible for energy production [93]. Mounting evidence continues to point toward mitochondrial dysfunction as an important factor in various neuropsychiatric conditions [94,95]. Elevated uric acid is emerging as a contributor of mitochondrial dysfunction, likely by increasing the burden of oxidative stress and interfering with normal enzymatic reactions [96]. In addition to playing a fundamental part in the production of energy, mitochondria are involved in a host of cellular functions, with implications for healthy neurotransmission and social behavior [97]. In a recent animal study, intentional elevations of blood uric acid resulted in higher brain levels or uric acid and increased hippocampal mitochondrial uric acid levels vs. control animals. The elevations in mitochondrial uric acid were not benign—reduced activity of mitochondrial enzymatic activity and ATP production was observed [98]. Recent human research points toward alterations in gene expression (mitochondrial autophagy-related genes) that can help explain mitochondrial dysfunction and elevated uric acid in schizophrenia [99].

It is also worth noting that neuroimaging research using fMRI has shown that higher uric acid levels are associated with neuropsychological impairments and changes in areas of the brain governing attention, executive function, reward, and motivation [100]. In a 12-year longitudinal study involving adults without a history of stroke, dementia, and/or Parkinsonism, higher uric acid levels were associated with subsequent reduced volumes of total brain, grey matter, white matter, grey matter in the hippocampus, and hippocampus [101]. Separate cohort research has produced similar results, with noted links between elevated blood uric acid and widespread disruptions in brain microstructural integrity [102]. Central nervous system findings support separate observations that uric acid increases oxidative stress and interferes with mitochondrial functioning in other cells and tissue in the periphery [103].

Although the preponderance of research has examined the relationship between anger/hostility and blood pressure from the perspective of emotion as the driver of hypertension, emerging research suggests that elevated blood pressure is also an independent driver of neuroticism [104]. Moreover, elevations in blood pressure are associated with ‘emotional dampening,’ a reduced responsiveness to both positive and negative emotions; this blood-pressure-related emotional dampening has been linked to risky behavior [105] and a reduced capacity for empathetic accuracy and perspective-taking [106]. Here, it is important to note that uric acid is a metabolic driver of elevated blood pressure, one that is often overlooked in adolescent health [107]. Research shows that allopurinol can lower blood pressure among adolescents [108], which suggests that further biobehavioral research is warranted.

Gout, which has long been connected to elevated uric acid levels, has been linked to significant increases in suicide risk [109]. Research focused on the biological differences between suicide by violent (e.g., gunshot) and nonviolent (e.g., overdose, poisoning) means has also uncovered a potential mechanistic link to uric acid and purinergic dysfunction. Longstanding research has linked suicide by violent means with a prior life course history of impulsivity and aggression [110]. In a recent study, researchers demonstrated that victims of violent suicides had a significantly up-regulated expression of genes involved in purinergic signaling, a finding that was not observed in control populations or in suicide victims who died by non-violent means [111]. Recent evidence suggests that elevated uric acid might be a candidate marker to differentiate between the mania (higher uric acid) and depressive (lower uric acid) phases of bipolar disorder [112].

For now, it is unclear why violent self-harm and a history of aggressive behavior might be related to the upregulation of gene expression related to purinergic signaling. However, it has long been recognized that purines (most notably ATP and adenosine) are responsible for critical extracellular communication [113]: adenosine maintains the functional properties of an inhibitory neurotransmitter; the blockage of adenosine receptors is associated with aggression [114]; and adenosine analogs inhibit fighting behavior [115]. Adenosine can be metabolized into uric acid, which suggests that rapid turnover in a vulnerable person might remove a potential inhibitor of aggression, adenosine, and add a possible chemical provocateur. In any case, allopurinol appears to benefit cases of refractory aggression, and neuropsychiatric conditions more broadly, by both reducing uric acid and allowing for greater adenosine availability [116]. It is also important to note that the mechanisms by which adenosine exerts anti-depressant and stress-resiliency properties appear to operate through the gut microbiome [117], a topic we turn to next.

## 5. Uric Acid and the Microbiome

Some of the most exciting advances in the mechanistic understanding of neuropsychiatric disorders involve discoveries related to the gut–brain–microbiota axis. Emerging research from bench science, coincident with epidemiological and intervention research, has provided clear evidence for a microbial role in cognition and behavior. So-called fecal transplant studies have demonstrated that aspects of donor behavior and brain function can be transferred to animals that are recipients of the donor fecal material. For example, the transfer of fecal material from human infants with antibiotic-induced dysbiosis (i.e., disturbance of the microbiome) to recipient lab animals leads to aggressive-like behavior, behavioral changes that do not occur with the transfer of microbiota from healthy infants [118]. Gut-microbiome-associated information is carried directly to the brain via the vagus and spinal nerves [119], and indirectly via microbe-influenced humoral signaling molecules (e.g., cytokines), neuropeptides, and hormonal messengers [120]. Since dietary choices and patterns influence the gut microbiota, microbiome sciences have bridged long-standing discussions of nutrition and aggression/antisocial behavior [121], as well as overall mental health [122]. It is also important to note that accumulating animal and human research demonstrates that early-life adverse experiences result in gut microbial dysbiosis [123].

Emerging research in the realm of metabolic health and type II diabetes demonstrates that gut microbes are involved in purine metabolism, and systemic uric acid levels appear to be regulated by the gut microbiome [124,125]. For example, elevated uric acid levels are associated with reduced microbial diversity and higher levels of select microbes that have been linked to chronic diseases [126]. Research has shown that targeting the gut microbiome with probiotics can reduce systemic uric acid levels [127,128,129,130,131,132].

Beneficial gut microbes appear to reduce the production of uric acid and enhance its elimination through the colon [133,134]. Indeed, a recent study showed that adding dietary fiber to animal chow had a positive influence on gut microbiota, while lowering both blood and cerebral levels of uric acid. These changes in the microbiome and physiology were accompanied by behavioral signs of decreased anxiety [135]. Additional research shows that fiber can increase microbial metabolites that otherwise limit xanthine oxidase activity, thereby reducing uric acid levels [136]. Moreover, the aforementioned fecal transplant method demonstrates that when fecal material from donor animals with high uric acid levels is transplanted into healthy recipients, uric acid levels rise [137]. Hints that gut microbes play a significant role in systemic uric acid levels emerged decades ago when it was reported that antibiotics can lower uric acid levels [138]. However, even in the early 2000s, the idea that gut microbes (and their manipulation though probiotics) can influence mental health was considered outlandish [139].

The recent findings on the microbiome and uric acid are intriguing because they help to explain the historical inconsistencies between dietary patterns purported to produce gout and elevated uric acid. In general, plant-based dietary patterns are associated with lower uric acid levels, while patterns rich in animal products are linked to higher levels of uric acid [140,141]. However, “plant-based” is a vague term lacking specificity in dietary choices. Research shows that plant-based diets cannot be painted with the same brush, and can easily translate into diets that are high in ultra-processed foods with added sugars, refined fats, emulsifiers, flavor enhancers, and other components that disturb the microbiome [142]. Indeed, ultra-processed packaged foods have been linked with elevated uric acid levels [143,144].

Since added sugars have the potential to elevate uric acid [145,146], much attention has been paid to increases in added sugars (especially fructose component and high-fructose corn syrup) as at least a partial explanation for the global increase in hyperuricemia [147,148]. However, pure fruit beverages (i.e., 100% juice with high levels of fructose) are not associated with elevated uric acid levels [149]. The consumption of pure fruit juices rich in phytochemicals is associated with improved mental outlook [150] and reduced anger and hostility [151], outcomes that appear to be mediated by beneficial influences on the gut microbiome. Flavonoids, and their metabolites formed through gut microbial fermentation, have been reported to lower systemic uric acid in adults [152,153]. In short, relationships between dietary patterns and blood/brain uric acid levels are more complex than previously appreciated, especially with the previously unrecognized influences of fiber and polyphenols at the diet–gut microbiota interface [154,155].

Taken as a whole, emerging evidence suggests that the gut microbiome plays a role in ‘transducing’ large-scale external forces, such as dietary patterns and their commercial determinants, as well as individual-level factors, in the production of chemicals (including uric acid) that are of relevance to brain and behavior [121]. Recently, courts in the United States and Europe have dismissed driving while intoxicated charges because the defendants were able to prove that significant elevations in blood alcohol were caused by an interaction between gut microbes and dietary carbohydrates [156]. That is, in the absence of oral alcohol consumption, defendants proved that gut microbial fermentation (microbes acting upon an oral carbohydrate load) was leading to significant internal alcohol production and systemic absorption at levels over legal limits. This condition, known as auto-brewery syndrome, essentially removes criminal intent, at least when an individual is unaware of their syndrome [156].

Auto-brewery has presented a clear challenge for the courts because alcohol is obviously not the only gut-mediated chemical that can potentially compromise cognition and increase susceptibility to reckless or antisocial behavior [157]. It has opened the door to what has been termed the legalome—microbiome and multi-omics science being applied in forensic and legal psychology [158]. Based on the research described above, uric acid is emerging as an important microbiome-mediated (i.e., legalome) compound, one that could influence criminal intent.

## 6. Biopsychosocial Context and Neurolaw Biomarkers

Undoubtedly, there is a need for more robust research, including evidence-based approaches that can test the idea that uric acid plays a causative role in behaviors that lead to justice involvement. At present, there is enough bench, preclinical, and epidemiological research to at least suggest that uric acid is more than a mere associative marker. Future directions in research and practice must evolve in tandem to harness the insights of biological criminology in a responsible manner. The human research connecting uric acid to cognition and behavior is intriguing, but remains limited by flaws that include small sample sizes, cross-sectional designs, and a lack of attention to acute and chronic mediators of uric acid. This latter consideration includes a long list of factors, including, but not limited to, diet [159], alcohol [160], tobacco/nicotine [161], medications [162], illicit drugs [163], the baseline microbiome [164], visceral fat/adiposity [165], existing metabolic and/or circulatory diseases [166], age [167], and psychological stress [168]. While epidemiological links between elevated uric acid and socioeconomic status remain equivocal [169], these mediators underscore that the psychosocial context and its relationship to the complexity of aggressive and antisocial behavior cannot be ignored.

The search for single biomarkers that are reliably connected to aggression, violence, and antisocial behavior has been wrought with difficulty and disappointment [170]. Historical findings in areas such as blood cortisol and testosterone have been conflicting, which has led to presumptions that biomarkers are unreliable. Many older studies ignored social and environmental contexts and lumped acute and chronic stress (and by extension, glucocorticoids) together. However, researchers have not abandoned the pursuit of biomarkers, and more recent evidence, with improved methodology, has provided a more nuanced relationship between cortisol and specific types of crime, including those associated with impulse control [171]. Higher blood testosterone levels have been associated (regardless of gender) with violence, callous–unemotional behavior, and impulsive antisocial activity [172,173]. Like uric acid, testosterone and cortisol can influence neurotransmitters [174]. It is interesting to note that higher testosterone and lower sex-hormone-binding globulin are associated with higher uric acid levels [175], and that exogenous estrogen is linked to lower (and exogenous testosterone with higher) uric acid levels [176,177]. Relationships between elevated cortisol and uric acid have also been noted, especially under stressful conditions [178].

Advances in behavioral endocrinology add to a larger frame that points toward the inclusion of multiple biomarkers. For example, the measurement of both testosterone and cortisol may provide more predictive value for aggression than either alone [179]. While individual inflammatory immune chemicals (e.g., cytokines) [180] have been linked with aggression, it is more likely that the measure of multiple immune chemicals—a peripheral immune inflammation index—will likely provide better predictive value [181]. The MAO-A gene, which encodes the neurotransmitter-modulating enzyme monoamine oxidase, showed promise in its initial connections to aggressive and violent behavior (hence the moniker “warrior gene”). However, a reliable relationship between single genes, including MAO-A, and violent behavior has not stood up to scrutiny under high-quality research designs [182]. Instead, a polygenic link to aggression, violence, and antisocial behavior will likely emerge [183] that can be linked with multi-omics investigations [184]. Taken as a whole, the emerging evidence suggests that the complexity of aggression and violence will be reflected in multiple, rather than single, biomarkers; here, uric acid might serve to increase the reliability of hormones, immune chemicals, and polygenic markers as part of the emerging concept of neuroprediction [185].

In order to move uric acid from an intriguing to a causal candidate, research methods will need to be improved, and several avenues of research demand attention:

o Longitudinal Cohorts: Following children from early life to adulthood to determine whether high uric acid levels, shaped by diet and adversity, predict future risk of aggression and justice involvement. These studies should examine key mechanistic pathways, including the following:
  - Blood–brain barrier integrity and its relationship to uric acid penetration.
  - Mitochondrial function and ATP production.
  - Inflammatory markers and their correlation with behavioral outcomes.
  - Gut microbiome composition and diversity.
  - Purinergic signaling patterns. This comprehensive approach would help clarify how early-life factors influence both biological vulnerabilities and behavioral trajectories.
o Randomized Trials: Testing whether interventions targeting uric acid (e.g., dietary changes, probiotics, xanthine oxidase inhibitors) tangibly reduce aggression in forensic or high-risk populations.
o Mechanistic Investigations: Using advanced neuroimaging and transcriptomics to clarify how hyperuricemia and purine metabolism affect prefrontal control, emotional regulation, and impulsivity. This includes the incorporation of uric acid into neuro-molecular investigations of criminal behavior [186].

## 7. Legal Context and Neuroprediction

“*When we reflect that a slight excess or deficiency of uric acid or ammonia in the blood shall make the same man at one time religious, moral, continent, and placable, and at another, irascible, unreasonable, licentious, and irreligious…we* [should] *forgive the offender seventy times seven times, rather than risk the infliction of an unjust punishment*” [187]

Arthur L. Wigan, MD, 1844

As evidenced by the quote above, the idea that uric acid and other metabolic chemicals should be considered by the courts is not a novel concept. The uric acid observations made by Louis Berman at Sing Sing prison in the 1930s [22] were overshadowed by his primary focus on endocrine dysfunction. However, they presaged contemporary discussions of how biological susceptibilities and social environments can intersect to foster aggressive or antisocial behavior. Modern studies confirm that hyperuricemia per se is insufficient to explain why all individuals offend. Yet, it may well amplify the likelihood of impulsivity or aggression, particularly under certain psychosocial conditions. Prenatal stress, early-life adversity, and environmental toxicants (such as lead) have each been shown to affect both uric acid metabolism [188,189,190] and the risk of antisocial outcomes [191], hinting at a biopsychosocial continuum rather than a one-dimensional cause. Indeed, elevated uric acid in psychiatric disorders may be a reliable biomarker of increased oxidative stress and lipid peroxidation, driven by psychosocial stressors [192].

As discussed in the introduction, justice-relevant advances in neurobiology are nested within neurolaw, a concept that challenges the prescientific assumptions (e.g., free will, near-universal levels of “willpower”, and blameworthiness) that otherwise underpin most legal systems [193,194]. Historically, legal systems have treated biological explanations of behavior with skepticism, often from fear of deterministic or reductionist claims. Yet, neurolaw approaches, informed by genetics, neuroscience, and microbiology, advocate for a refined integration of such data into adjudicative and rehabilitative processes [195]. If the research connecting uric acid (or any biological marker identified through omics technologies) and aggression/antisocial behavior becomes increasingly robust, it will force the following considerations:

1. Mens Rea and Capacity: Courts traditionally apply a binary lens to culpability [196]. Biological influences, however, need not absolve defendants of responsibility; rather, they help situate the defendant’s neurobiology within contextual stressors and environmental burdens, thereby informing sentencing and rehabilitative strategies. Evidence that elevated uric acid—whether influenced by individual metabolic vulnerability, dietary patterns, and/or stress—can diminish impulse control suggests that a purely “willful” model of wrongdoing may be oversimplified. Consider that the prescientific notions of blameworthiness and willpower are, in form and function, not dissimilar to outdated notions of blame and shame that have historically been directed at persons living with obesity. While obesity is clearly not criminal behavior, it is worth pointing out the absurdity of the idea that the massive and relatively sudden rise in obesity rates over the last four decades is a product of an instantaneous loss of willpower among global populations. Obesity has little to do with willpower [197] and much to do with commercial determinants of health [198] as they intersect with the microbiome and brain circuitry [199] and, in keeping with our topic here, uric acid production [200]. Yet, courts still cling to a willpower philosophy of human behavior [194].

2. Sociolegal Context: Individuals from disadvantaged communities often endure chronic stress, suboptimal dietary options (particularly ultra-processed foods), and disproportionate exposure to toxins [201]. These stressors facilitate hyperuricemia and related inflammation [202]. Instead of relying solely on punitive measures, justice systems could incorporate nutritional, medical, or stress-modulation interventions, acknowledging that social inequities contribute to biological vulnerabilities and thus to behavioral outcomes [203]. In order to avoid the pitfalls of “biological determinism” (which essentially ignores the social drivers of disease and behavior), there is a need to consider the social exposome in which hyperuricemia might sit. Is uric acid simply a downstream marker of marginalization, disadvantage, and the social, psychosocial, socioeconomic, sociodemographic, local, regional, and cultural aspects of behavior [204]? In the context of neuroprediction (even with the combined input of neuroimaging and biological markers, of which uric acid and omics metabolites may be a part), it is also important to consider the ways in which ‘labeling’ an individual as biologically dangerous could have far-reaching ripple effects that might include unjust social exclusion and discrimination [205].

3. Therapeutic Jurisprudence: In matters where aggression or impulsivity appear linked to biological markers, courts and correctional facilities could consider therapeutic [206] as opposed to purely punitive approaches. For instance, if robust screening and clinical judgment identify hyperuricemia as a relevant factor, it may be prudent to offer interventions known to lower uric acid, as well as produce collateral health benefits. These might include dietary counseling [207], select probiotics [164], exercise [208], psychoeducational interventions [209], yoga [210,211], and/or mindfulness practices [212]. Similarly, the success of allopurinol in attenuating aggression in specific psychiatric populations underscores the potential value of targeted metabolic interventions [213,214].

4. Policy Implications: From a neurolaw perspective, neither biology nor environment alone suffice to explain all antisocial conduct. Instead, rigorous biopsychosocial assessments can inform policy. Included in this will be the need for screening and assessment tools that might consider objective metabolic markers (including uric acid levels) for individuals whose charges involve violent conduct, with careful attention given to informed consent and privacy. The obvious policy implications include matters of sentencing and rehabilitation. Here, there may be a need to codify sentencing guidelines that entertain the possibility that—where scientifically warranted—biological interventions might mitigate recidivism. Since uric acid and other legalome factors can be downstream manifestations of social and commercial determinants of health, community-level interventions may be warranted. These might include broader nutritional policies that address food deserts, ultra-processed diets, and environmental toxin exposures, with the aim of reducing systemic risk factors for hyperuricemia and downstream aggression.

## 8. Conclusions

The history of uric acid in criminological inquiry shows that seemingly esoteric physiological factors—largely disregarded by mid-century scientists—may hold genuine relevance for understanding aggression. Present-day research firmly situates uric acid within a broader network of stress responses, microbiome functioning, and dietary habits [157]. The available research, despite its aforementioned limitations, suggests that uric acid, as a potential biomarker of risk, is worthy of further research and close scrutiny. From a neurolaw perspective, integrating this knowledge can inform more humane, scientifically grounded policies that recognize the interplay between biology and environment. The history of the quest for single biomarkers of interest to criminology is fraught with disappointment. It is more likely that uric acid, even if high-quality research were to provide support for its role as a candidate marker of risk, will provide complementary information, and add to other markers (e.g., hormonal, endocrine, immune) and neuroimaging measures as part of a neuroprediction mosiac.

Crucially, such biological integration should avoid a worldview that emphasizes reductionist determinism [215]. Even if uric acid in concert with metabolomics and neuroimaging/electroencephalogram findings were to reliably predict risk, this would not equate to inevitable destiny at the individual level; risk can be mitigated through public health measures, individual medical interventions, and rehabilitative frameworks that encourage meaningful transformation. Wherever the emerging research on uric acid and the legalome might land, we must attend to both biology and context, and uphold the twin goals of justice—accountability for harmful behavior and an evidence-based pathway that points toward rehabilitation.

## Data Availability

No new data were created.

## Abbreviations

The following abbreviations are used in this manuscript:

HPA	Hypothalamic–pituitary–adrenal (HPA) axis
ATP	Adenosine triphosphate
fMRI	Functional magnetic resonance imaging
